# A nurses’ perspective on life satisfaction of parents of children with type 1 diabetes mellitus

**DOI:** 10.3389/fendo.2026.1834784

**Published:** 2026-07-21

**Authors:** Anna Stefanowicz-Bielska, Ewa Kobos, Alicja Szewczyk, Agnieszka Olińska, Małgorzata Rąpała, Martyna Piechotka-Klonowska

**Affiliations:** 1 Division of Internal and Pediatric Nursing, Institute of Nursing and Midwifery, Faculty of Health Sciences with the Institute of Maritime and Tropical Medicine, Medical University of Gdansk, Gdansk, Poland; 2 Department of Pediatrics, Diabetology and Endocrinology, University Clinical Center in Gdansk, Gdansk, Poland; 3 Polish Federation for Education in Diabetology, Warsaw, Poland; 4 Department of Development of Nursing, Social and Medical Sciences, Faculty of Health Sciences, Medical University of Warsaw, Warsaw, Poland; 5 Department of Endocrinology and Diabetology, Diabetes Clinic, Children’s Memorial Health Institute, Warsaw, Poland; 6 Endocrinology and Diabetology Pediatric Department VI with Sub-department of Rheumatology, Prof. Dr Stanislaw Popowski Regional Specialized Children’s Hospital, Olsztyn, Poland; 7 Department of Pediatric Surgery, Marciniak Hospital, Wroclaw, Poland

**Keywords:** child, life satisfaction, parents, pediatric chronic illness, type 1 diabetes mellitus

## Abstract

**Introduction:**

Parental involvement in a child’s self-monitoring and treatment, including challenges encountered during treatment, influences the life satisfaction of caregivers of children with type 1 diabetes mellitus (T1DM).

**Aim:**

This study aimed to assess the level of life satisfaction among parents of children with T1DM and to examine the impact of selected sociodemographic and medical factors on life satisfaction.

**Materials and methods:**

The study was conducted between September 1^st^, 2024, and March 1^st^, 2025, using a questionnaire designed by the author and the standardized Satisfaction with Life Scale (SWLS). Participants were parents of children aged 2 to 18 with a diagnosis of T1DM for more than one year, who were receiving care at four Diabetes Centers in the Mazovian, Pomeranian, and Warmian-Masurian voivodeships in Poland.

**Results:**

A total of 327 parents of children with T1DM participated in the study. The mean ages of mothers and fathers were 41.5 ± 6.6 years and 44.1 ± 7.1 years, respectively, and the average age of the children was 11.8 ± 3.9 years. The mean duration of diabetes was 5.47 ± 3.62 years. The median SWLS sten score was 6.0 (4.0÷7.0). Higher life satisfaction was observed among professionally active fathers, parents living in nuclear families, and parents reporting a very good financial situation. Parents of children with T1DM who consulted a psychologist, psychotherapist, or psychiatrist reported lower life satisfaction.

**Conclusions:**

The life satisfaction of parents of children with T1DM was average. The parents of children with T1DM who have lower life satisfaction more likely to seek psychological support. The study demonstrated the influence of the roles of family members (mother vs. father), the father’s employment status, family structure, and the family’s financial status on life satisfaction. Independent sociodemographic factors were parent/caregiver and family financial status. Given the complexity of T1DM management, multidisciplinary support is essential for both children and their families. Efforts should be made to protect the mental health of children with T1DM and their parents. Facilitated access to specialist care should be prioritized. Nurses should develop family-centered care plans and work to reduce factors that negatively affect the life satisfaction of children and their families. The current parental life satisfaction score is important in providing daily care for children with T1DM. If the parental life satisfaction is low, the family should be provided with psychological support. Nurses should actively cooperate with psychologists, psychotherapists, and social workers in caring for children with T1DM, and should also inform caregivers on the current methods of support for families of children with chronic diseases. Up-to-date parental life satisfaction should be assessed during follow-up visits with educational nurses (diabetes educators) at the Diabetes Clinic. The development and implementation of a screening questionnaire would be a valuable component of nursing care planning for children with T1DM and their parents, as it would enable the rapid identification of the needs of both children and their parents (e.g., regarding education, emotional support, and caregiving). This would facilitate comprehensive, family-centered, and personalized care, while helping to prevent caregiver burnout, and improve the quality of family functioning in their home environment. These measures would support the individualization of care plans, enable early crisis intervention, and improve communication between parents and healthcare professionals. Healthcare professionals play a key role in identifying parental difficulties and ensuring specialized care for those most in need. A holistic and systemic approach that addresses both physical and mental health is crucial for improving the outcomes, self-monitoring results, and treatment of T1DM in children and adolescents, as well as for improving the quality of life of children and their parents.

## 1 Introduction

Type 1 diabetes mellitus (T1DM) is an autoimmune disorder in which the immune system attacks insulin-producing ß cells in the pancreas, causing a deficiency in insulin (1).

T1DM is one of the most prevalent chronic diseases in children, although its prevalence varies considerably across regions worldwide (1).

589 million adults (20-79 years) are living with diabetes worldwide (2). Type 1 and type 2 diabetes are the two main types, with type 2 diabetes accounting for the majority (>85%) of total diabetes prevalence (3). The total number of adults with diabetes is predicted to rise to 853 million by 2050 (2). As of 2024, an estimated 9.15 million people globally have a clinical diagnosis of T1DM, of whom 22.3% (2.04 million) live in low- and middle-income countries (4).

Approximately 1.81 million individuals (19.8%) are younger than 20 years, 6.28 million (68.6%) are aged 20 to 59 years, and 1.06 million (11.8%) are aged 60 years or older. In 2024, there were 503,000 newly diagnosed cases of T1DM across all age groups, with nearly half (219,000) occurring in children and adolescents younger than 20 years (4). In Poland, the number of adults aged 20–79 years living with diabetes is estimated at 3,098.6 in 1,000s, while the number of individuals with T1DM across all age groups is 130,658.0 (5). Globally, 1,211,900 children and adolescents younger than 20 years have T1DM (6). In Poland, there are 20,182.6 individuals with T1DM aged 0-19 years. The highest increase in T1DM diagnoses was observed in children aged 0-4 years (7, 8).

The etiology of T1DM remains unknown and is believed to result from complex interactions between multiple genetic and environmental factors. Three stages of T1DM have been described: stage 1 is characterized by autoimmunity and normoglycemia, stage 2 involves progression to presymptomatic dysglycemia, and stage 3 is symptomatic disease requiring insulin therapy (1).

The management of T1DM is a demanding and complicated process, requiring regular subcutaneous insulin administration, accurate carbohydrate counting of meals, systematic monitoring of blood glucose levels in capillary blood and/or interstitial fluid, and regular physical activity (1).

The goal of diabetes management in children and adolescents is the prevention of acute and chronic complications, while achieving and maintaining normal physical development, including appropriate height, body weight, and body composition (percentile values), as well as age and sex-appropriate pubertal development, and ensuring a good quality of life for the child and their family.

According to the Polish Society of Diabetology, the key parameters associated with a reduced risk of vascular complications in the treatment of T1DM include:

- Time in range (TIR; percentage of time within the individual target glucose range) ≥80%, coefficient of variation (CV) <36%, and HbA1c (glycated hemoglobin) ≤6.5%, while minimizing episodes of hypoglycemia and maintaining good quality of life. During remission and when using automated insulin delivery systems, a narrower target range may be considered, defined as time in tight range (TITR; 70–140 mg/dL). The recommended targets are TITR >55% and CV <33%.
- Total cholesterol levels: <170 mg/dl (<4.4 mmol/l), low-density lipoprotein (LDL) cholesterol <100 mg/dl (<2.6 mmol/l), triglycerides <100 mg/dl (<1.1 mmol/l).
- Blood pressure: <90^th^ percentile for age, sex, and height (for individuals ≥13 years of age <120/80 mm Hg).
- Body mass index (BMI) <85^th^ percentile for age and sex (9).

Appropriate management of T1DM, including effective treatment and patient self-management, can prevent and delay the onset of many diabetes-related complications (1).

Treatment of T1DM requires active engagement of both the child and their parents in ongoing care and self-management. Parental involvement in the process of the child’s treatment and self-management, as well as challenges encountered during disease management, may influence the life satisfaction of parents of children with T1DM.

This study aimed to assess the level of life satisfaction among parents of children with T1DM and to examine the impact of selected sociodemographic and medical factors on life satisfaction.

## 2 Materials and methods

### 2.1 Study design, setting and participants

The cross-sectional survey was conducted between 1st September 2024 and 1st March, 2025.

**Diagram 1:**
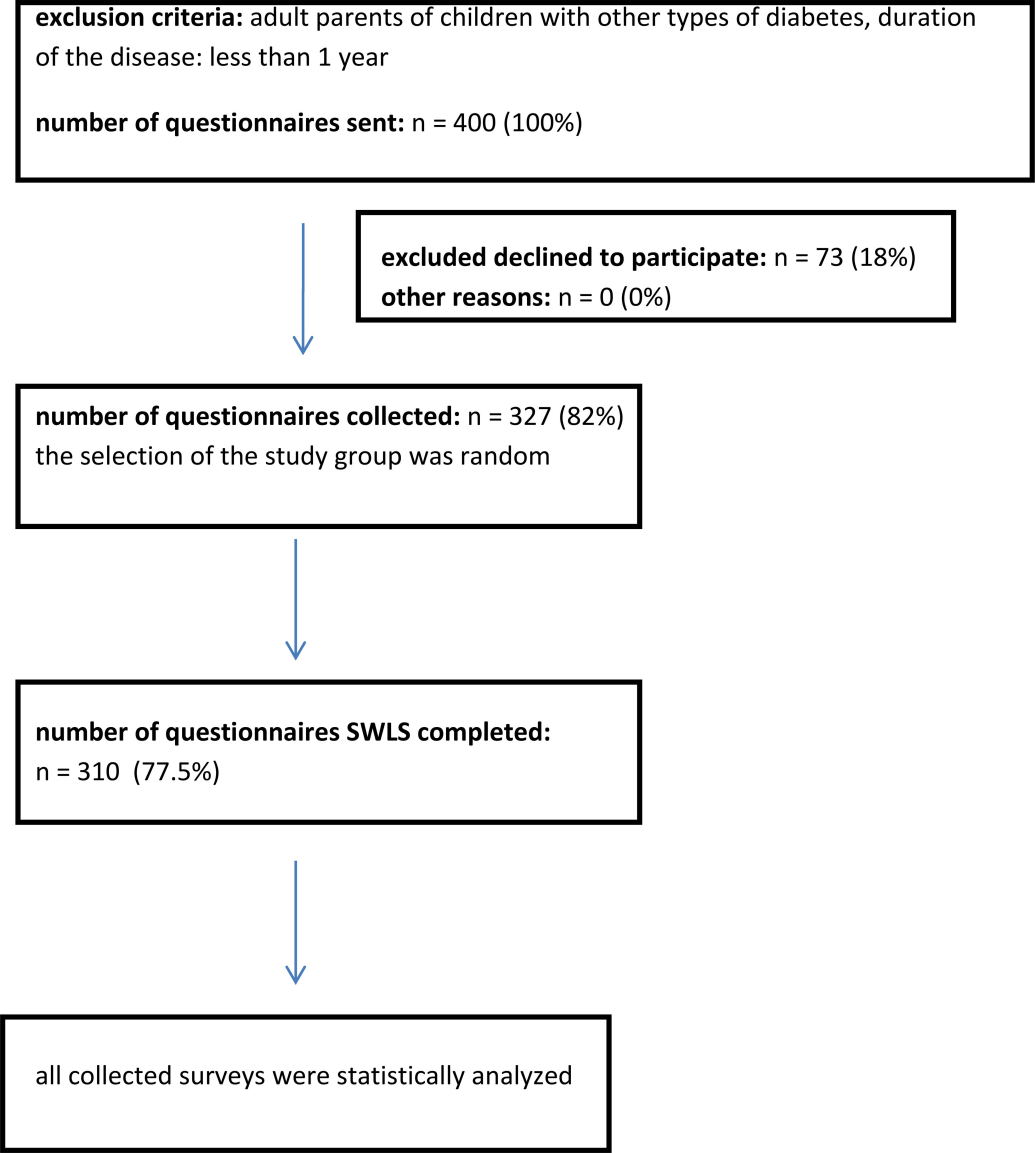
Consort diagram.

Study participants were parents of children aged 2 to 18 with a diagnosis of T1DM for more than one year, who were receiving care at four Diabetes Centers in the Mazovian, Pomeranian, and Warmian-Masurian voivodeships in Poland. A total of 400 questionnaires were sent (Diagram 1.). Only one parent of one child completed the survey. The parent participation was random. The authors used consecutive sampling. The researchers defined specific inclusion and exclusion criteria and personally contacted every single parent who crossed in the Diabetes Centers and agreed to fill the survey.

### 2.2 Methods

The study was conducted using a survey designed by the author and the standardized Satisfaction with Life Scale (SWLS), developed by Diener, Emmons, Larsen, and Griffin from the Faculty of Psychology at the University of Illinois (adapted to Polish conditions by Z. Juczyński) (10).

To assess life satisfaction, a basic scale adapted to Polish conditions and suitable for use by nurses was selected.

#### 2.2.1 Self-authored survey

The self-authored survey consisted of two parts.

The first part of the survey contained information on the study and an invitation to participate in the study, while the second part consisted of questions on sociodemographic and medical data. According to the recommendations of the Polish Society of Diabetology – 2026, HbA_1_C ≤6.5% is considered to be one of the indicators of metabolic balance (9).

#### 2.2.2 The satisfaction with life scale

The SWLS contains five statements: 1. In most ways my life is close to my ideal, 2. The conditions of my life are excellent, 3. I am satisfied with my life, 4. So far, I have gotten the important things I want in life and 5. If I could live my life over, I would change almost nothing.

Each participant assessed the extent to which each of the statements applied to their life so far. Each statement is assessed on a scale of 1 to 7, where 1 signifies “strongly disagree”, and 7 signifies “strongly agree”.

The Cronbach alpha coefficient of the SWLS was 0.81 in a study of 371 adults, indicating satisfactory reliability. Test-retest reliability, evaluated in a group of 30 adults over a six-week interval, had a stability coefficient of 0.86. In the original version of the scale, Cronbach’s alpha was 0.87, and the test-retest correlation, over a two-month period was slightly lower at 0.82 (10, 11).

The total result of the SWLS is calculated as the sum of all five statements. The result of the test translates into the level of life satisfaction. The range of results is between 5 and 35 points, which is a raw score. The higher the score, the higher the sense of satisfaction with life (10).

Raw scores were converted to sten scores, which were used to interpret the results. A sten score of 1-4 is interpreted as a low result, while a score of 7-10 is considered a high result. Sten scores of 5 and 6 are average results (10).

### 2.3 Data collection

The researchers provided parents with the surveys and questionnaires. Participation in the study was voluntary. The directors of the healthcare facilities provided consent for the study. Approval for the study was granted by the Independent Bioethics Committee for Scientific Research of the Medical University of Gdansk (KB/275/2024).

### 2.4 Statistical methods

The results were subjected to statistical analysis. For quantitative variables, the number of cases (N), mean, standard deviation (SD), median, range (minimum-maximum), and lower and upper quartiles (25Q÷75Q) were calculated for all groups.

Depending on the distribution, quantitative data were presented as:

1. mean ± SD, for normally distributed variables;
2. median and interquartile range M (25Q÷75Q), for non-normally distributed variables.

Qualitative variables were presented as absolute values and percentages (%).

Normality of distribution was assessed using the Shapiro-Wilk test, and homogeneity of variances was evaluated with Levene’s test.

Differences between independent groups were analyzed using one-way analysis of variance (ANOVA) for normally distributed variables with homogeneous variances. For variables with non-normal distribution or heterogeneous variances, the Mann–Whitney U test (for two groups) or the Kruskal–Wallis test (for three or more groups) was used. When comparing three or more groups, multiple two-sided comparisons were performed as appropriate.

Independent sociodemographic factors were identified using backward step-wise regression analysis.

A p-value of <0.05 was considered statistically significant. Statistical analyses were conducted using Statistica software, version 13.3 (TIBCO Software Inc.).

## 3 Results

### 3.1 Patient characteristics

A total of 327 parents of children with T1DM participated in the study, including 267 (82.7%) mothers, 50 (15.5%) fathers, and 6 (1.8%) other caregivers (foster parents, a grandfather, a grandmother, an uncle, and an aunt). In four surveys, respondents didn’t indicate the type of caregiver. The mean age of the mothers, fathers, and children was 41.5 ± 6.6 years, 44.1 ± 7.1 years, and 11.8 ± 3.9 years, respectively. The mean duration of diabetes was 5.47 ± 3.62 years (range, 1 year-15 years). The mean concentration of glycated hemoglobin (HbA1c) in children was 7.4% ± 1.54% (range, 5%-18.3%).

Most participants were married mothers aged 41-50 years, with a university education, living in urban areas, and professionally active in non-physical occupations that did not require shift work. These caregivers typically lived in nuclear families, reported a good financial situation, and did not require additional financial assistance. Most children in these families were aged 13-18 years, had siblings, and attended elementary school. The majority of children had been diagnosed with T1DM for 2 to 5 years, were treated with tubed insulin pumps, used bolus calculators, and regularly used continuous glucose monitoring (CGM) systems. Most surveyed parents/caregivers regularly counted carbohydrate exchanges or grams of carbohydrates in meals. Notably, 81.7% parents/caregivers did not keep a traditional self-monitoring diary, and only 31.5% of parents maintained a digital self-monitoring diary. Approximately half of all parents/caregivers and children with T1DM did not attend appointments with a diabetes nurse educator (50.8%), and most participants did not schedule appointments with a dietitian (75.5%), psychologist (73.8%), psychotherapist (87.2%), or psychiatrist (86.5%) at the diabetes clinic.

### 3.2 Life satisfaction

The SWLS was completed by 310 respondents. Completion of the SWLS by parents was voluntary. The results of the SLWS were analyzed.

The median sten scores was 6.0 (4.0÷7.0).

### 3.3 The influence of selected sociodemographic and medical factors on life satisfaction

The results of the influence of sociodemographic and medical factors on life satisfaction are presented in **Tables 1**–**3**.

**Table 1:** Sociodemographic factors and life satisfaction of parents of children with T1DM, who participated in the SWLS study.

Sociodemographic factors		N (%)	Life satisfaction – raw score	Life satisfaction – stens
Parent/caregiver	mother	256 (84.8%)	20.0 (16.0÷24.0)	5.0 (4.0÷7.0)
	father	46 (15.2%)	23.0 (19.0÷25.0)	6.0 (5.0÷7.0)
p			0.008**	0.013**
Sex of child	female	146 (48%)	20.9 ± 6.1	5.66 ± 2.14
	male	158 (52%)	20.6 ± 5.3	5.53 ± 1.9
p			0.703*	0.569*
Age of mother	≤ 30 years	16 (5.3%)	21.5 (18.5÷28.0)	5.50 (5.00÷8.00)
	31-40 years	126 (42%)	20.0 (15.0÷24.0)	5.00 (4.00÷7.00)
	41-50 years	134 (44.7%)	22.0 (17.0÷25.0)	6.00 (4.00÷7.00)
	≥ 51 years	24 (8%)	21.0 (17.0÷24.0)	5.00 (4.00÷7.00)
p			0.127***	0.154***
Age of father	≤ 30 years	5 (1.7%)	24.0 (19.0÷28.0)	7.00 (5.00÷8.00)
	31-40 years	82 (27.7%)	20.5 (17.0÷24.0)	5.50 (4.00÷7.00)
	41-50 years	167 (56.6%)	21.0 (17.0÷25.0)	6.00 (4.00÷7.00)
	≥ 51 years	41 (13.9%)	20.0 (16.0÷24.0)	5.00 (4.00÷7.00)
p			0.676***	0.621***
Age of child	preschool age – 4-6 years old	30 (9.8%)	19.5 (17.0÷25.0)	5.00 (4.00÷7.00)
	school age – 7-12 years old	136 (44.4%)	21.0 (16.0÷25.0)	6.00 (4.00÷7.00)
	adolescence – 13-18 years old	140 (45.8%)	21.0 (17.0÷24.0)	6.00 (4.00÷7.00)
p			0.918***	0.856***
Place of residence	small town	36 (11.8%)	21.1 ± 6.6	5.67 ± 2.23
	medium-sized town	72 (23.6%)	21.2 ± 5.7	5.79 ± 2.07
	city	80 (26.2%)	20.7 ± 5.7	5.60 ± 1.96
	village	117 (38.4%)	20.3 ± 5.4	5.40 ± 1.94
p			0.760*	0.617*
Education of mother	elementary school	4 (1.3%)	20.0 (17.0÷21.5)	5.50 (4.50÷6.00)
	high school	106 (34.5%)	20.0 (16.0÷23.0)	5.00 (4.00÷6.00)
	vocational	34 (11.1%)	23.0 (19.0÷27.0)	6.00 (5.00÷8.00)
	university	163 (53.1%)	21.0 (17.0÷25.0)	6.00 (4.00÷7.00)
p			0.080***	0.105***
Education of father	elementary	13 (4.3%)	20.0 (17.0÷23.0)	5.00 (4.00÷6.00)
	high school	112 (37.3%)	20.5 (16.0÷24.0)	5.50 (4.00÷7.00)
	vocational	68 (22.7%)	21.0 (17.0÷24.0)	6.00 (4.00÷7.00)
	university	107 (35.7%)	21.0 (17.0÷25.0)	6.00 (4.00÷7.00)
p			0.353***	0.328***
Siblings	yes	247 (82.1%)	21.0 ± 5.8	5.70 ± 2.04
	no	54 (17.9%)	19.8 ± 5.5	5.26 ± 1.91
p			0.156*	0.151*
Type of school (child)	elementary school	183 (63.3%)	20.0 (16.0÷24.0)	5.00 (4.00÷7.00)
	high school	52 (18%)	22.5 (17.5÷24.0)	6.00 (4.50÷7.00)
	technical college	39 (13.5%)	20.0 (17.0÷23.0)	5.00 (4.00÷7.00)
	vocational school	9 (3.1%)	24.0 (20.0÷25.0)	7.00 (5.00÷7.00)
	preschool	6 (2.1%)	17.5 (17.0÷22.0)	4.50 (4.00÷6.00)
p			0.328***	0.269***
Marital status of parents	married	250 (82.5%)	21.0 (17.0÷25.0)	6.00 (4.00÷7.00)
	informal relationship	25 (8.3%)	20.0 (17.0÷24.0)	5.00 (4.00÷7.00)
	widowed	2 (0.6%)	23.5 (20.0÷27.0)	6.50 (5.00÷8.00)
	single	26 (8.6%)	19.5 (15.0÷23.0)	5.00 (4.00÷6.00)
p			0.208***	0.159***
Professional status - mother	employed	193 (63.3%)	21.0 ± 5.4	5.69 ± 1.94
	unemployed	112 (36.7%)	20.3 ± 6.2	5.40 ± 2.17
p			0.286*	0.226*
Professional status - father	employed	283 (95.3%)	21.0 (17.0÷25.0)	6.00 (4.00÷7.00)
	unemployed	14 (4.7%)	17.5 (14.0÷20.0)	4.50 (3.00÷5.00)
p			0.037**	0.046**
Type of work - mother	non-physical	148 (72.5%)	21.0 (17.0÷25.0)	6.00 (4.00÷7.00)
	physical labor	51 (25%)	21.0 (16.0÷24.0)	6.00 (4.00÷7.00)
	both	5 (2.5%)	19.0 (18.0÷24.0)	5.00 (5.00÷7.00)
p			0.855***	0.954***
Type of work - father	non-physical	114 (40.6%)	20.0 (17.0÷25.0)	5.00 (4.00÷7.00)
	physical labor	160 (56.9%)	21.0 (17.0÷24.0)	6.00 (4.00÷7.00)
	both	7 (2.5%)	23.0 (19.0÷26.0)	5.00 (5.00÷7.00)
p			0.636***	0.796***
Shift work (day/night) - mother	yes	22 (7.1%)	22.0 (17.0÷24.0)	6.00 (4.00÷7.00)
	no	288 (92.9%)	21.0 (17.0÷24.5)	6.00 (4.00÷7.00)
p			0.412**	0.434**
Shift work (day/night) - father	yes	49 (15.8%)	21.0 ± 5.7	5.69 ± 1.99
	no	261 (84.2%)	20.7 ± 5.7	5.57 ± 2.03
p			0.763*	0.724*
Family structure	nuclear	267 (87.8%)	21.0 ± 5.7	5.68 ± 2.01
	single-parent	37 (12.2%)	19.0 ± 5.4	4.97 ± 1.94
p			0.043*	0.046*
Family financial status	very good	81 (26.4%)	23.0 (18.0÷27.0)	6.00 (5.00÷8.00)
	good	219 (71.3%)	20.0 (17.0÷24.0)	5.00 (4.00÷7.00)
	poor	7 (2.3%)	14.0 (9.0÷15.0)	3.00 (1.00÷4.00)
p			0.000***	0.000***
Additional financial assistance	yes	98 (32.8%)	20.2 ± 7.0	5.36 ± 2.07
	no	200 (67.2%)	21.0 ± 5.0	5.71 ± 1.99
p			0.213*	0.157*

*ANOVA; **Mann–Whitney U test; ***Kruskal–Wallis test.

**Table 2:** Glycated hemoglobin concentration, duration of disease, chronic disease in children and life satisfaction of parents of children with T1DM, who participated in the SWLS study.

Medical factors		N (%)	Life satisfaction – raw score	Life satisfaction – stens
Glycated hemoglobin concentration - child	≤6.5%	64 (27.8%)	20.3 ± 5.8	5.41 ± 2.04
	>6.5%	166 (72.2%)	20.9 ± 5.6	5.67 ± 2.01
p			0.444*	0.367*
Duration of disease – child	1 year	17 (6.2%)	21.0 (16.0÷25.0)	6.00 (4.00÷7.00)
	2-5 years	149 (54%)	20.0 (16.0÷24.0)	5.00 (4.00÷7.00)
	6-10 years	74 (26.8%)	22.0 (19.0÷25.0)	6.00 (5.00÷7.00)
	over 10 years	36 (13%)	20.5 (17.0÷23.0)	5.50 (4.00÷6.00)
p			0.428***	0.335***
Hypothyroidism - child	yes	29 (9.4%)	20.1 ± 5.8	5.38 ± 2.08
	no	281 (90.6%)	20.8 ± 5.7	5.62 ± 2.02
p			0.529*	0.537*
Celiac disease - child	yes	11 (3.5%)	22.0 (17.0÷24.0)	6.00 (4.00÷7.00)
	no	299 (96.5%)	21.0 (17.0÷25.0)	6.00 (4.00÷7.00)
p			0.816**	0.906**
Bronchial asthma - child	yes	13 (4.2%)	19.0 (15.0÷22.0)	5.00 (4.00÷6.00)
	no	297 (95.8%)	21.0 (17.0÷25.0)	6.00 (4.00÷7.00)
p			0.105**	0.103**
Depression - child	yes	13 (4.2%)	15.0 (11.0÷19.0)	4.00 (2.00÷5.00)
	no	297 (95.8%)	21.0 (17.0÷25.0)	6.00 (4.00÷7.00)
p			0.002**	0.002**

*ANOVA; **Mann–Whitney U test; ***Kruskal–Wallis test.

**Table 3:** Medical factors and life satisfaction of parents of children with T1DM, who participated in the SWLS study.

Medical factors		N (%)	Life satisfaction – raw score	Life satisfaction – stens
Insulin pen	yes	50 (16.1%)	21.0 (17.0÷28.0)	6.00 (4.00÷8.00)
	no	260 (83.9%)	21.0 (17.0÷24.0)	6.00 (4.00÷7.00)
p			0.228**	0.184**
Tubed insulin pump	yes	221 (71.3%)	21.0 (17.0÷24.0)	6.00 (4.00÷7.00)
	no	89 (28.7%)	21.0 (16.0÷26.0)	6.00 (4.00÷7.00)
p			0.546**	0.446**
Tubeless insulin pump	yes	24 (7.7%)	23.0 (15.5÷25.0)	6.00 (4.00÷7.00)
	no	286 (92.3%)	21.0 (17.0÷24.0)	6.00 (4.00÷7.00)
p			0.893**	0.753**
Bolus calculator	yes	198 (63.9%)	20.8 ± 5.6	5.59 ± 1.96
	no	112 (36.1%)	20.7 ± 5.9	5.62 ± 2.13
p			0.873*	0.916*
Glucose monitor	yes, regularly	112 (36.1%)	21.6 ± 5.7	5.89 ± 2.02
	yes, not regularly	127 (41%)	19.9 ± 5.7	5.31 ± 2.01
	no	71 (22.9%)	21.0 ± 5.6	5.66 ± 2.01
p			0.068*	0.078*
Continuous glucose monitoring (CGM)	yes, regularly	233 (80.3%)	21.0 (17.0÷24.0)	6.00 (4.00÷7.00)
	yes, not regularly	22 (7.6%)	20.5 (17.0÷23.0)	5.50 (4.00÷6.00)
	no	35 (12.1%)	21.0 (17.0÷26.0)	6.00 (4.00÷7.00)
p			0.923***	0.973***
Traditional paper self-monitoring diary	yes	51 (18.3%)	20.8 ± 6.2	5.67 ± 2.13
	no	228 (81.7%)	20.8 ± 5.7	5.62 ± 2.03
p			0.984*	0.879*
Digital self-monitoring diary	yes	87 (31.5%)	21.3 ± 5.9	5.80 ± 2.07
	no	189 (68.5%)	20.6 ± 5.9	5.54 ± 2.07
p			0.373*	0.325*
Regular calculation of carbohydrate exchanges or grams of carbohydrates	yes	224 (76.2%)	20.7 ± 5.8	5.57 ± 2.02
	no	70 (23.8%)	20.9 ± 5.5	5.60 ± 2.02
p			0.820*	0.905*
Child with parent/caregiver appointments with a dietitian	yes, regularly	25 (8.8%)	20.0 (16.0÷23.0)	5.00 (4.00÷6.00)
	yes, not regularly	45 (15.7%)	20.0 (17.0÷23.0)	5.00 (4.00÷6.00)
	no	216 (75.5%)	21.0 (17.0÷25.0)	6.00 (4.00÷7.00)
p			0.717***	0.629***
Child with parent/caregiver appointments with a psychologist	yes, regularly	38 (12.9%)	19.4 ± 5.7	5.11 ± 2.10
	yes, not regularly	39 (13.3%)	19.2 ± 6.1	5.03 ± 2.10
	no	217 (73.8%)	21.2 ± 5.6	5.76 ± 1.98
p			0.004*	0.033*
Child with parent/caregiver appointments with a psychotherapist	yes, regularly	15 (5.2%)	18.0 (12.0÷20.0)	5.00 (3.00÷5.00)
	yes, not regularly	22 (7.6%)	21.0 (15.0÷24.0)	6.00 (4.00÷7.00)
	no	251 (87.2%)	21.0 (17.0÷25.0)	6.00 (4.00÷7.00)
p			0.017***	0.018***
Child with parent/caregiver appointments with a psychiatrist	yes, regularly	19 (6.6%)	18.0 (14.0÷23.0)	5.00 (3.00÷6.00)
	yes, not regularly	20 (6.9%)	21.5 (14.5÷25.0)	6.00 (3.50÷7.00)
	no	249 (86.5%)	21.0 (17.0÷25.0)	6.00 (4.00÷7.00)
p			0.050***	0.029***
Child with parent/caregiver appointments with a diabetes nurse educator	yes, regularly	90 (31%)	21.0 (16.0÷23.0)	6.00 (4.00÷6.00)
	yes, not regularly	53 (18.2%)	22.0 (18.0÷25.0)	6.00 (5.00÷7.00)
	no	148 (50.8%)	20.0 (17.0÷25.0)	5.00 (4.00÷7.00)
p			0.524***	0.500***
Parent/caregiver appointments with a diabetes nurse educator	yes, regularly	39 (12.8%)	21.7 ± 5.7	5.9 ± 2.09
	yes, not regularly	88 (28.9%)	19.9 ± 5.7	5.26 ± 1.91
	no	178 (58.3%)	21.1 ± 5.6	5.72 ± 2.01
p			0.144*	0.131*
Parent/caregiver appointments with a psychologist/psychotherapist	yes, regularly	38 (12.9%)	19.2 ± 5.9	5.00 ± 2.14
	yes, not regularly	39 (13.3%)	19.5 ± 5.9	5.14 ± 2.07
	no	217 (73.8%)	21.3 ± 5.5	5.79 ± 1.95
p			0.045*	0.035*
Parent/caregiver appointments with a psychiatrist	yes, regularly	18 (5.9%)	19.0 (15.0÷23.0)	5.00 (4.00÷6.00)
	yes, not regularly	14 (4.6%)	18.0 (16.0÷23.0)	5.00 (4.00÷6.00)
	no	272 (89.5%)	21.0 (17.0÷25.0)	6.00 (4.00÷7.00)
p			0.122***	0.086***
Parent/caregiver appointments with a dietitian	yes, regularly	31 (10.3%)	22.0 ± 4.8	5.94 ± 1.82
	yes, not regularly	55 (18.2%)	19.8 ± 5.3	5.18 ± 1.78
	no	216 (71.5%)	21.0 ± 5.8	5.70 ± 2.05
p			0.188*	0.151*

*ANOVA; **Mann–Whitney U test; ***Kruskal–Wallis test.

The results demonstrated that fathers (stens – mother 5.00 (4.00÷7.00) vs father 6.00 (5.00÷7.00), p=0.013), professionally active fathers (stens – employed 6.00 (4.00÷7.00) vs unemployed 4.50 (3.00÷5.00), p=0.046), parents living in nuclear families (stens – nuclear 5.68 ± 2.01 vs single-parent 4.97 ± 1.94, p=0.046), and parents reporting a very good financial situation (stens – very good 6.00 (5.00÷8.00) vs good 5.00 (4.00÷7.00) vs poor 3.00 (1.00÷4.00), p=0.000) had a higher level of life satisfaction (**Table 1**).

In contrast, parents of children with T1DM who consulted a psychologist (stens – yes, regularly 5.11 ± 2.10 vs yes, not regularly 5.03 ± 2.10 vs no 5.76 ± 1.98, p=0.033), psychotherapist (stens – yes, regularly 5.00 (3.00÷5.00) vs yes, not regularly 6.00 (4.00÷7.00) vs no 6.00 (4.00÷7.00), p=0.018) or psychiatrist (stens – yes, regularly 5.00 (3.00÷6.00) vs yes, not regularly 6.00 (3.50÷7.00) vs no 6.00 (4.00÷7.00), p=0.029) reported lower life satisfaction (**Table 3**).

Independent sociodemographic factors were identified using backward step-wise regression analysis:

Life satisfaction – raw score = f (parent/caregiver, professional status – father, family structure, family financial status).

Independent sociodemographic factors were parent/caregiver and family financial status R = 0.30, R^2^ = 0.09, adj R^2^ = 0.09, F(2.296)=14.912, p<0.00000.

Life satisfaction – stens = f (parent/caregiver, professional status – father, family structure, family financial status).

Independent sociodemographic factors were parent/caregiver and family financial status R = 0.32, R^2^ = 0.10, adj R^2^ = 0.09, F (2.296)=16.462, p<0.00000.

## 4 Discussion

T1DM may influence the quality of life of children and adolescents, and their parents. Parents caring for children with T1DM encounter many physical, mental, social, financial, and spiritual problems.

This study aimed to assess the level of life satisfaction among parents of children with T1DM and to examine the impact of selected sociodemographic and medical factors on the life satisfaction of parents of children with T1DM.

In our study, the median raw scores and sten scores of the SLWS were 21.0 (17.0÷24.0) and 6.0 (4.0÷7.0), respectively.

4.2% of children were reported to have depression. Parents of children who did not have depression had higher life satisfaction; however, these findings are difficult to interpret due to the small sample size. Additionally, the parents of children with T1DM, who have lower life satisfaction more likely to seek psychological support. However, no measures of depression, anxiety, or psychological distress were performed. Therefore, these findings are challenging to interpret.

According to Whittemore et al., an estimated 33.5% of parents reported feelings of stress at the time of their child’s diagnosis, whereas 19% of parents reported feelings of stress 1–4 years after diagnosis (12). The emotional responses reported by parents depend on the level of diabetes control. When the disease is metabolically well-controlled, families most commonly report feelings of confidence and balance; in contrast, poor metabolic control is associated with feelings of uncertainty (13). Over time, diabetes and its treatment become an integral part of the family’s daily routine, even when blood glucose level imbalances impact family life to various degrees; hence, life satisfaction varies (14, 15). Managing meal preparation, family conflict related to diabetes, insulin administration, monitoring glucose levels in capillary blood or interstitial fluid, as well as concerns about long-term complications, are frequent sources of stress experienced by caregivers, particularly mothers (16). High stress levels associated with a child’s diabetes have been identified as a risk factor for anxiety and depression in mothers. Depression is one of the most prevalent mental conditions observed in mothers (17).

Our study demonstrated that fathers reported higher life satisfaction than mothers. Overall, 26.2% of parents had consulted a psychologist and/or psychotherapist. Parents of children with T1DM who did not consult a psychologist or psychotherapist reported higher life satisfaction, most likely reflecting more effective coping with their child’s illness.

Maternal depression has been shown to affect a child’s metabolic control (18). Children, whose mothers exhibit more severe symptoms of depression, have a higher risk of hospitalization due to acute diabetes-related complications (19, 20).

In the Baudino’s study, First STEPS (*Study of Type 1 in Early childhood and Parenting Support*), evaluated a stepped-care behavioral intervention comprising increasingly intensive intervention steps (peer parent coach, cognitive-behavioral counseling, consultations with diabetes educator and psychologist) based on need. In this trial were parents of young children newly diagnosed with T1DM. In children, HbA1c levels and parental depression symptoms decreased compared to baseline values after the interventions (21).

One Turkish study assessed the factors influencing life satisfaction among 293 parents of children with T1DM aged 3–18 years, who were treated in a pediatric clinic. Data were collected using the *Adult Life Satisfaction Scale* (ALSS). The mean ALSS score was 69.39 ± 11.61. Statistically significant differences in total ALSS scores were observed according to parental education level, self-reported parents health status before and after the child’s T1DM diagnosis, income level, professional activity, the impact of the disease on daily responsibilities, perceived ability to cope with self-monitoring and treatment of T1DM, and the availability of additional support in childcare (p<0.05). Higher life satisfaction was reported by parents with higher education (p<0.05), good health status before and after the child’s T1DM diagnosis (p<0.001), income exceeding expenses (p<0.001), professionally active parents (p<0.001), effective coping with self-monitoring and treatment (p=0.001), and access to childcare support (p<0.05) (22).

Faulkner et al. measured the quality of life (QOL) of 30 parents of children aged 10 to 12 years and 26 parents of children aged 13 to 18 years. None of the children had any known developmental delays, and all had been diagnosed with T1DM at least one year before the study. Parental QOL was measured using the Parent Diabetes Quality of Life Questionnaire (PDQOL), which comprises three subscales: Diabetes Life Satisfaction, Disease Impact, and Disease-related Worries. No statistically significant differences in life satisfaction were observed according to race, sex of the child or parent, or parental education level. However, within the Diabetes Life Satisfaction subscale, a statistically significant difference was identified based on parental marital status (p=0.033). Divorced parents were less satisfied with life compared with cohabiting couples. However, it must be noted that the sample of divorced parents was substantially smaller (n=9) than that of married parents (n=44) (23).

In the Faulkner’s study the mean levels of life dissatisfaction were higher in the group of parents with lower income compared to parents with average or higher incomes. However, this result was not statistically significant. No significant correlations were found between parents’ QOL scores and the duration of diabetes, insulin injections, and blood glucose measurements using a glucometer. There was, however, a positive correlation between life satisfaction and metabolic control (R = 0.364, p=0.009) (23).

In our study, no association was found between children’s glycated hemoglobin levels and parents’ life satisfaction. Similarly, the child’s age had no significant effect on parental life satisfaction. However, professionally active fathers, parents living in nuclear families, and parents reporting a very good financial situation demonstrated significantly higher life satisfaction. We analyzed independent sociodemographic factors which should have influence on parentals’ life satisfaction. For backward step-wise regression was taken parent/caregiver, professional status – father, family structure, family financial status (these were statistically significant factors in univariate analysis). Parent/caregiver and family financial status turned out to be the only independent factors, which we examined.

A study conducted in Turkey evaluated the caregiving burden and quality of life of 106 mothers of children with T1DM. The mothers had a moderate level of caregiving burden, which was associated with a negative impact on their quality of life (24).

da Costa et al. evaluated the Health-Related Quality of Life (HRQOL) of 96 adolescents aged 10–19 years living in the metropolitan region of midwestern Brazil, who had been diagnosed with T1DM for at least one year. Most adolescents had been diagnosed more than three years earlier and were receiving more than three insulin injections per day. The majority of participants reported at least one episode of hypo- or hyperglycemia within the last month (93%). Moreover, the mean HbA1c level was 9.6%. Adolescents who were diagnosed with T1DM at an earlier age had a lower HRQOL (25).

In our study, children with T1DM had a mean HbA1C level of 7.4% ± 1.54% (minimum – 5%; maximum – 18.3%). Overall, 72.2% of children had HbA1C>6.5%. Children’s HbA1c values were not associated with parental life satisfaction.

Continuous subcutaneous insulin infusion (CSII) is a safe and effective method of insulin therapy in young children with T1DM, with benefits such as improved glycemic control (lower HbA1c levels), higher quality of life, and greater life satisfaction, compared with multiple daily injections. The use of insulin pumps may improve the quality of life of children with T1DM and their families by providing greater flexibility in meal planning and reducing the burden associated with daily injections (26, 27). In our study, 71.3% of children used a tubed insulin pump, 7.7% used a tubeless insulin pump, and 16.1% used an insulin pen. The majority of children were treated with insulin pump therapy. However, the study did not demonstrate an association between the method of insulin delivery and parental life satisfaction.

A group of researchers from Kuwait and the United Kingdom evaluated the impact of switching from multiple daily insulin injections to insulin pumps on glycemic control and daily functioning among children/adolescents with T1DM (5–17 years; N = 34) and their parents (N = 38). The majority of parents (N = 32) and children/adolescents (N = 30) reported that glycemic control was easier to maintain within the target range with pumps than with injections. Participants generally found insulin pump devices easy to use and more acceptable than injections. Both parents and children/adolescents reported increased lifestyle flexibility and improved participation in home, school, and social activities whilst maintaining glycemic control. The greatest difficulties were reported during the initial phase of pump use. Overall, insulin pump therapy conferred benefits in terms of glycemic control, general well-being, enabling young people to be more in control of their condition and lead more normal lives (28).

Mednick et al. assessed the life satisfaction among 22 children with T1DM and their parents during the initiation of continuous subcutaneous insulin infusion therapy, as well as quality of life at a later follow-up stage. Overall, participants reported high satisfaction with insulin pump use, particularly noting the flexibility associated with sleeping and eating (29).

Our study did not demonstrate a significant correlation between the type of insulin therapy or self-monitoring method and the life satisfaction of parents of children with T1DM. Overall, 79% of children used continuous subcutaneous insulin infusion, and 87.9% used CGM.

A child’s T1DM diagnosis presents substantial challenges for parents as it necessitates significant changes in the daily functioning of the entire family. Children/adolescents and their family members experience psychological difficulties following diagnosis with T1DM and undergo a prolonged and demanding adaptation phase. In addition to the impact of T1DM on the family’s quality of life (professional limitations, financial burden, treatment costs), the diagnosis itself generates negative emotions in children and their parents (30).

Diagnosis of T1DM in a child is often associated with anger, denial, fear, and depression from the parents. The aim of the Italian study was to improve parents’ adaptation to the diagnosis of diabetes of their child. Sixty-two parents (29 mothers, 33 fathers) of 36 children with T1DM (mean age=11.3-3.3 years; diabetes duration > 1 year; HbA1c=57 ± 11 mmol/mol) participated in a three-day educational working group pilot intervention study. Relaxing technique, diaphragmatic breathing, and guided visualization were used by 2 psychologists and 1 pediatric endocrinologist.

Three months after the intervention, both parents reported a reduction in the “difficult child” subscale of the PSI-SF (Parenting Stress Index Short Form) and increased scores of social functioning of the SF-36 (Health Survey Short Form-36). DRD (Diabetes-Related Distress) score was significantly reduced in mothers, while the “parental distress” subscale of the PSI-SF (Parenting Stress Index Short Form) was significantly improved in fathers. This weekend-based parent group intervention seems to reduce stress and improve social functioning of parents of children and adolescents with T1DM (31).

Living with T1DM remains a challenge for children and their families. Young patients depend heavily on their caregivers, who provide daily support with self-monitoring and treatment.

When caring for a child with a chronic illness, families may experience mental, emotional, physical, social, and financial problems related to the additional costs of treatment and medical devices, increased caregiving time, sleep deprivation, and stigmatization (22).

Practical implications:

1. The current parental life satisfaction score is important in providing daily care for children with T1DM. If the parental life satisfaction is low, the family should be provided with psychological support. The findings suggest that the nurses should actively cooperate with psychologists, psychotherapists, and social workers in caring for children with T1DM, and should also inform caregivers on the current methods of support for families of children with chronic diseases. Up-to-date parental life satisfaction should be assessed during follow-up visits with educational nurses (diabetes educators) at the Diabetes Clinic.
2. The authors propose that the development and implementation of a screening questionnaire would be a valuable component of nursing care planning for children with T1DM and their parents, as it would enable the rapid identification of the needs of both children and their parents (e.g., regarding education, emotional support, and caregiving). This would facilitate comprehensive, family-centered, and personalized care, while helping to prevent caregiver burnout, and improve the quality of family functioning in their home environment. These measures would support the individualization of care plans, enable early crisis intervention, and improve communication between parents and healthcare professionals.

## 5 Limitations of the study

Some of the study limitations are:

- the use of a general scale.

This scale was chosen as it is adapted to Polish conditions and serves as a basic tool to assess life satisfaction. The SWLS is not disease-specific. It does not evaluate parental distress, anxiety, or depression. The use of a general scale does not precisely specify the specific burden of caregivers, which influenced the obtained research results and that why this constitutes a limitation of the work. Future studies should be extended to assess the caregiving burden of parents of children with T1DM. This will be the next research of the authors’ work.

- a small sample size and surveying only four Polish regions.

Life satisfaction depends on many different factors. A limited number of factors influencing parents’ life satisfaction were examined, primarily the impact of the disease and treatment principles, as well as the life limitations resulting from these factors.

- there were no questions about the parental/caregiver’s medical history in the self-authored questionnaire.
- the participants had children in the different age and with different duration of the disease.
- the authors of the study didn’t assess the incidence of depressive disorders and caregiver burden in parents/guardians of children with T1DM.

## 6 Conclusions

Parental life satisfaction of children with T1DM is average. The parents of children with T1DM who have lower life satisfaction more likely to seek psychological support. The study demonstrated the influence of the roles of family members (mother vs. father), the father’s employment status, family structure, and the family’s financial status on life satisfaction. Independent sociodemographic factors were parent/caregiver and family financial status. Due to the complexity of T1DM treatment, multidisciplinary support is critical for both children and their families. Efforts should be made to protect the mental health of children with T1DM and their parents. Facilitated access to specialist care should be prioritized. Nurses should develop family-centered care plans and work to reduce factors that negatively affect the life satisfaction of children and their families.

Healthcare professionals play a key role in identifying parental difficulties and ensuring specialized care for those most in need. A holistic and systemic approach that addresses both physical and mental health is crucial for improving the outcomes, self-monitoring results, and treatment of T1DM in children and adolescents, as well as for improving the quality of life of children and their parents.

## Data Availability

The datasets presented in this article are not readily available because data available on request due to privacy restriction. The corresponding author has the database. Requests to access the datasets should be directed to Anna Stefanowicz-Bielska; ania-stefanowicz@gumed.edu.pl.
